# A qualitative analysis of men’s involvement in maternal and child health as a policy intervention in rural Central Malawi

**DOI:** 10.1186/s12884-018-1669-5

**Published:** 2018-01-19

**Authors:** Elizabeth Mkandawire, Sheryl L. Hendriks

**Affiliations:** 1 Institute for Food, Nutrition and Wellbeing, Information Technology Building, Room 3-37, University of Pretoria, Pretoria, 0002 Republic of South Africa; 2 Institute for Food, Nutrition and Wellbeing, Information Technology Building, Room 3-34, University of Pretoria, Pretoria, 0002 Republic of South Africa

**Keywords:** Men, Gender, Nutrition, Policy, Malawi

## Abstract

**Background:**

Men’s involvement in maternal and child health presents an opportunity for the advancement of maternal and child nutrition as men often play a key role in decision-making particularly regarding women’s reproductive health. While most research on men’s involvement in maternal and child health has focused on men’s participation in antenatal care, this study focuses specifically on men’s involvement in maternal and child nutrition. The purpose of the study is to explore how men’s involvement is conceptualised in rural Central Malawi, highlighting the key factors influencing men’s involvement in maternal and child health.

**Methods:**

Focus group discussions and in-depth interviews were conducted with 26 informants and 44 community members. Critical policy was used as the theoretical framework to inform the analysis of research findings.

**Results:**

In this study, we identified several factors that facilitate men’s involvement in maternal and child health, but we also identified several barriers. Facilitators of men’s involvement included: recognition by men of the impact of their involvement, pride, advocacy, incentives and disincentives and male champions. Barriers included socio-cultural beliefs, stigmatisation and opportunity costs. The study also found that there were several limitations that had unintended consequences on desired programme outcomes. These included: discriminating against women, marginalisation of married women and reinforcing men’s decision-making roles.

**Conclusion:**

The study findings highlight the importance of involving men in maternal and child health for improved nutrition outcomes. We emphasise the need for nutrition policy-makers to be aware that gender dynamics are changing. It is no longer just women who are involved in nutrition activities, therefore policy-makers need to revise their approach to ensure that they consider men’s role in nutrition.

## Background

Men’s involvement in maternal and child health has been on the development agenda for over two decades. However, in policy practice, it has for the most part been overlooked and excluded from policy design. Nutrition policies place significant emphasis on women, particularly pregnant and lactating women. Drawing from evidence indicating that empowering women can lead to improved child nutrition outcomes and in recognising the importance of addressing gender inequality, nutrition policy-makers have often targeted women as beneficiaries of such policy interventions [1]. Recent policy initiatives, for example, have been directed to the first thousand days of a child’s life wherein women have been the primary focus of policy interventions. Proponents of this development approach posit that if women and children consume the necessary dietary requirements from conception up until a child’s second birthday, under-nutrition can be reduced [2]. While empowering women is indeed crucial, the narrow focus on women [3], overlooks women’s limited decision-making power [4] and the gendered dynamics of resource allocation [5].

International agreements such as the Beijing Declaration and Platform for Action [6] and the Rome Declaration [7] require states to ensure that both gender and nutrition respectively, be mainstreamed into all policies. Gender mainstreaming refers to the integration of men and women’s concerns in the design and implementation of policies and programmes at all levels. Gender power relations are often overlooked in the design of policies, leading to a narrow focus on women because of the genuinely urgent need to address women’s issues [8]. In the case of nutrition policy, emphasis is placed on women, and men continue to be excluded owing to socio-cultural determinants dictating that pregnancy, child care and nutrition are the realm of women [9, 10]. When men are excluded, women may face challenges in negotiating for resources to purchase food that is nutritious. A study by Singh et al. [11] found that when men are involved in maternal and child health, one of the roles they identify with is ensuring that their partners are eating properly. In this current study, we analyse how gender normative assumptions, such as nutrition being women’s responsibility, have shaped policies and how these assumptions have been deconstructed by the emergence of the men’s involvement agenda. The study contributes to literature that informs the integration of gender in nutrition policies. In reporting our findings, we highlight the implications of including men when integrating gender into nutrition policy.

### Men’s involvement

The definition of men’s involvement varies depending on the context in which it is applied. According to Ditekemena et al. [12] men’s involvement is considered to be men’s participation in HIV testing during pregnancy. Alio et al. [13] offer a different perspective from the Western context, by suggesting that men’s involvement is “…being accessible (e.g. present, available), engaged (e.g. cares about the pregnancy and the coming child, wants to learn more about the process), responsible (e.g., is a caregiver, provider, protector), and maintaining a relationship with the woman carrying the child regardless of their own partnership status.”.

The literature on men’s involvement in maternal and child health suggests that men’s involvement in antenatal clinics results in positive maternal and child health outcomes [11]. For example, it increases women’s attendance of antenatal clinics, compliance with breastfeeding, family planning and the uptake of Prevention of Mother-to-Child-Transmission of HIV/AIDS (PMTCT) [14–16]. In their study Kalembo et al. (2013) [15] found that women who attended antenatal care were more likely to agree to an HIV test, deliver their babies at the hospital and complete postnatal follow-up treatment. Kululanga et al. [9], highlight how the use of incentives, male peer initiatives and sensitisation campaigns served as tools for encouraging men to participate in antenatal clinics.

Socio-cultural institutions that prescribe gender roles to men and women create barriers to men’s involvement in maternal and child health and nutrition [9, 17]. Men who divert from socio-culturally proscribed gender roles by, for example engaging in child care or cooking, are often stigmatised and sanctioned by their fellow men [4, 18]. Food and nutrition are typically considered women’s responsibility, subsequently men do not realise that they have a role to play in nutrition and thus may not be involved [19, 20].

In low-income countries, men’s involvement has focused on men’s involvement in PMTCT [4, 12, 15, 16, 19, 20]. Less attention has been paid to men’s involvement in maternal and child nutrition, where men’s decision-making role has significant implications for maternal and child health outcomes. This study explores the implications of men’s involvement for integrating gender in nutrition policy. The specific aims of the study were to develop an understanding of how men’s involvement is defined in rural Central Malawi and what hinders and facilitates men’s involvement.

### Safe motherhood in Malawi

Safe motherhood initiatives were introduced in Malawi in 2000 to reduce the incidence of maternal mortality which was 984 per 100,000 live births in 2004 and 675 per 100,000 live births in 2010 [21]. An important part of this initiative is to involve men, given their household decision-making role in relation to women’s access to healthcare. In some cases, women not only need to obtain resources but also permission from their husbands in order to access healthcare [22].

As part of the Malawi safe motherhood initiative, maternity waiting homes (MWH) were established as a way of increasing women’s access to trained health care professionals at a health facility for delivery. Maternity waiting homes are built in close proximity to a health facility. These waiting homes help to reduce the risk of giving birth at home and increase the likelihood of delivery by a qualified health care professional [23]. In Ntcheu, the Traditional Authorities have established bylaws that prevent women from giving birth outside of a health facility. If women contravene these bylaws, they are subject to paying a fine [23]. Bylaws on safe motherhood have increased the number of women giving birth at health facilities at the hands of a skilled birth attendant. In Ntcheu, the 2016 Malawi Demographic and Health Survey reported that 91% of women gave birth at a health facility [24] compared to 37% in the Central Region in 2004 [25]. The bylaws established by Traditional Authorities are communicated to people in the community by word of mouth but also through other media channels like the radio.

While policies are established at the national and government level, bylaws are often established at the local level by community leaders in the form of Traditional Authorities or village headmen or headwomen. However, the Constitution does not bestow legislative power on Traditional Authorities. Therefore, these bylaws are not legally binding. Traditional leaders vary in their level of involvement on issues pertaining to the health and nutritional well-being of the communities they oversee. A number of noteworthy traditional leaders are well known for developing bylaws that have had a positive impact on maternal child health and nutrition well-being in their communities. For example, Chief Kwataine, a renowned chief from Ntcheu district at the Traditional Authority level, has become a champion of safe motherhood in recent years.

### The research context

Malawi is located in sub-Saharan Africa, south of the equator. The country is divided into three regions, the North, South and Central region. Within these three regions, there are 28 districts, which are further sub-divided into Traditional Authorities, a customary form of leadership responsible for oversight of several villages. The villages are in turn governed by village headmen or headwomen who are under the leadership of the Traditional Authority. The village is the smallest administrative unit [26]. Traditional Authorities are managers of customary land, custodians of customary law and guardians of tradition and culture.

In the last two decades, Malawi has experienced negative climatic events that have increased in frequency, intensity and magnitude, resulting in reduced agricultural productivity. Even moderate droughts have had a severe impact on food security for families in rural Malawi [27]. With the floods that took place in early 2015 and the consequent drought in 2016, caused by El Nino, food security in Malawi has only worsened. This is evidenced by 37% of children under five suffering from stunting with only 8% of infants 6–23 meeting the minimum acceptable diet [25]. Minimum acceptable diet refers to consumption from at least four food groups, four times a week. The Malawi Demographic and Health Survey (2016) estimates that, as of 2015, infant mortality was at 42 deaths per 1000 live births [25]. During the period 2008–2012, under-nutrition could be attributed to 23% of Malawi’s child mortality [28].

### Study location

This study was conducted in one Traditional Authority in Ntcheu, a district located in the Central Region of Malawi. With a population of 499,936 [29], 88% of households in Ntcheu engage in subsistence farming, with only 5 % earning wages through formal employment. Most households (61%) consume the food that they produce [28].

Although the main road through Ntcheu is tarred, most communities are far removed from the main road. Ewing et al. [30] suggest that communities living in hard to reach areas do not access health care facilities frequently because of the financial costs associated with transportation. Also, for rural dwellers involved in subsistence farming, access to healthcare often involves long distance travel. Consequently, they lose a whole day of farm work, which has important implications for crop production. In Ntcheu, 60% of the population reported that distance to the health facility constrained access to health care services [25].

Health Surveillance Assistants were first introduced in 1973 as cholera assistants to mediate the cholera outbreak. In 1998, the government recognised the need to bring health services closer to the community. Cholera assistants were renamed Health Surveillance Assistants and their work was broadened to include a range of health care services such as conducting bi-monthly mobile clinics for postnatal health checks and child immunisations. In the study area, approximately eight Health Surveillance Assistants are available for a population of 8715.

## Methods

### Study design

This study was executed as part of the Feed the Future Food Security Policy Innovation Lab [31] to understand the implications of men’s involvement in maternal and child health on the integration of gender in nutrition policy and explored the opportunities that exist for nutrition policy-makers within the context of men’s involvement in maternal and child health. In attempting to understand the phenomenon of men’s involvement as a policy intervention, we employed qualitative research methods using a critical policy framework. Because of the transdisciplinary nature of nutrition, we included multiple stakeholders (men, women, decision-makers from various sectors, traditional leaders as well as health care providers) to enhance the credibility of the study through the triangulation of opinions and perspectives.

Focus groups and individual interviews were conducted. The use of these two methods, along with the use of two different sample populations – informants and community members – provided a deeper understanding of men’s involvement in maternal and child health. To create a distinction between the two groups of participants, the non-state and state actors were referred to as the informants and the target population for policy implementation was referred to as community members. The data from the individual interviews was used to corroborate the data from the focus group discussions.

The informants all signed written consent forms prior to participating in the interviews or focus groups. Given the limitations in formal education in the community, consent to participate was recorded digitally by voice recording. A tape recorder was used capture each participant’s consent. Participants were given a number which they would state and then confirm that they consented to be part of the study. For example, “I am number … and I consent to participate in the study”. At the start of the session, the researchers emphasised that participation was voluntary and participants were not obliged to participate and could withdraw from the discussion at any time should they wish to do so, without any penalty.

### Focus group discussions

Focus group discussions were used to obtain perspectives of how men’s involvement in maternal and child health was understood in Malawi. Liamputtong [32] suggests that focus group discussions are valuable for eliciting community perspectives on various issues. The interactive nature of the focus group discussions enabled participants to challenge each other or present different personal experiences of men’s involvement in maternal and child health. This offered validation on the extent of convergence or divergence of themes related to men’s involvement. Discussions lasted for an hour to two hours. All focus group discussions were conducted in the communities from which the participants came and were held at the health centre or at a local community gathering place. The discussions were conducted once large groups had dispersed at the clinic to reduce distractions. The Health Surveillance Assistants, nurses and a community contact person ensured that discussions were not interrupted by making the community aware of our presence and the purpose of our visit. They also intercepted curious individuals who may have interfered with the discussions.

The community members were initially selected through purposive and then through convenience sampling, dependent on their availability at the time of the focus group discussion. The Health Surveillance Assistants were referred to us by the District Health Office. All eight Health Surveillance Assistants were interviewed. They assisted us in selecting focus group discussion participants at the clinic. At the maternity waiting homes, the nurses helped identify participants. In the community, the Traditional Authority identified a key informant to assist us during our field work. She helped to select men and women from the community to participate in the focus group discussions. A total of 63 participants were interviewed, 26 were informants and 37 were community members. The majority of participants were women (*N* = 44).

Seven focus group discussions were conducted with informants and community members. Three mixed-sex focus group discussions were conducted with the informants: one with eight Health Surveillance Assistants, another with six village headmen and women and the third with five officials from an NGO. Four focus group discussions were conducted with community members from three communities in rural Central Malawi. Two of these focus group discussions were conducted with women, one was conducted with men and one combined both women and men.

The discussions were facilitated in the local language, Chichewa by one of the female authors and an experienced female facilitator who are both fluent in Chichewa. The facilitator is an experienced qualitive researcher and skilled at minimising participant bias during interviews. She was able to create trust and rapport amongst the participants, providing a conducive environment for open discussion. The other facilitator provided support and moderated the discussion. The lack of a male facilitator for the men’s focus group discussions is recognised as a limitation. The researchers counted the number of times men and women spoke during the focus group discussions to determine if men’s participation was hindered by this. In all of the interviews women were slightly more vocal than men. The exception was the interview with eight Health Surveillance Assistants, where men responded 31 times compared to 21 responses form women. The composition of this group was skewed in favour of men because of the limited number of female Health Surveillance Assistants in the area. Men were engaged in the discussion and the researchers had no reason to believe that the lack of a male facilitator hindered or biased the open discussions.

While we recognise that there are constraints in conducting mixed-sex focus group discussions, these focus groups were useful to investigate gender perspectives. Having separate groups might have created a situation where men say one thing and women say another. But having men and women in the same group helped each group reach a consensus on the probable pathways to shift gender dynamics in favour of men’s increased involvement in maternal and child nutrition. Having separate gender discussion would be gender accommodating rather than transformative in nature [33].

We recognise that women can be marginalised in mixed sex focus group discussions. The power dynamics existent in society wherein men have more power over women can sometimes create a situation where women defer to men’s opinions. We took measures to ensure that women participated freely in the discussions. First, unlike much research into gender dynamics, the focus groups for this study did not analyse gender dynamics. Where current gender power dynamics would constrain honest discussions in mixed groups, these discussions sought to identify and negotiate pathways to change dynamics through reaching a consensus between the sexes. Second, three single-sex focus group discussions were conducted and the mixed-sex focus group discussions were used to triangulate the data from these discussions. Third, both men and women were consulted on participating in these mixed groups prior to participation. They had no concern about participating. Fourth, the ratio of women to men in these mixed-group discussions was deliberately skewed, with higher participation of women. Fifth, in all but one group, the participants were of an equal professional level. Sixth, participants were not asked to reflect on their personal experiences, but discussed possible pathways to change gender dynamics.

The mixed-sex focus group discussions converged with the single-sex focus group discussions, leading us to believe that mixed-sex focus group discussions did not hinder the study. There is an added benefit of corroborating the findings of the single-sex groups with a mixed group discussion. We also used the discussion as an opportunity to observe gender dynamics between professionals who are responsible for implementing gender policies. Understanding these interactions has implications for the development of policy.

### Individual interviews

Eighteen individual interviews were conducted with informants using an interview guide which outlined the key research issues and topics to be explored. Individual interviews were conducted for some of the informants’ group because it was difficult to schedule their participation in the focus groups but we needed their perspectives on the topic. Participants in the informants’ group were initially selected through purposive sampling following a stakeholder mapping process. Stakeholders in the Department of Nutrition, HIV and AIDS were identified. These informants referred the researchers to other stakeholders. As a result, snowball sampling was used.

These individual interviews included discussions with the District Health Officer; the Environmental Health officer; officers from the Ministry of Gender, Children, Disability and Social Welfare, the Ministry of Education, Science and Technology and the Department of Nutrition, HIV and AIDS as well as representatives from donor agencies, NGO and community members. Engaging with various groups and using a variety of research approaches such as focus group discussions and individual interviews enabled corroboration of data.

### Data analysis

All interviews were transcribed and Chichewa transcripts were translated into English. The transcripts were reviewed and notes were made for each transcript. The data analysis included manually organising the data into the three core themes of facilitators, barriers and limitations of men’s involvement. A table was developed of the sub-themes in each of the core themes (see Table 1). Once all the transcribed data were categorised into themes, the information was again reviewed, taking note of the frequency with which a certain theme occurred. The authors made sure to reflect on data that did not converge. For example, not all participants agreed that men were involved in maternal and child health. These opinions were taken into consideration in reporting the results. Quotations from the transcripts have been used to illustrate the sub-themes identified during the analysis. The informants’ and community members’ responses were analysed separately.

**Table 1:** Themes and sub-themes identified of men’s involvement in maternal and child health

Themes	Facilitators	Barriers	Limitations
Sub-themes	Men’s recognition of the benefits of participation	Socio-cultural beliefs	Discriminating against women
	Pride	Stigmatisation	Reinforcing men’s decision-making power
	Advocacy	Opportunity costs	
	Incentives and disincentives as provided by health care providers and Traditional Authorities		
	Encouragement from male champions		

## Results

Demographic data were collected for the community members’ group. Most participants in this group engaged in subsistence farming and *ganyu* to sustain their livelihoods. The mean age of participants was 37 with ages of participants ranging from 19 to 80 years. The average education of the participating community members was seven completed years of schooling, ranging from those with no education to participants who had obtained a secondary school certificate. Except for one women who was pregnant with her first child, all the community members had children.

We identified five main facilitators of men’s involvement: (i) men’s recognition of the benefits of participation; (ii) pride; (iii) advocacy; (iv) incentives and disincentives as provided by health care providers and Traditional Authorities; and (v) encouragement from male champions. We identified facilitators as factors that led to the renegotiation of socio-cultural barriers that have prevented men’s participation. Three barriers to men’s involvement were identified, including: (i) socio-cultural beliefs; (ii) stigmatisation and (iii) opportunity costs. Two limitations were identified in terms of men’s involvement including: (i) discriminating against women and (ii) reinforcing men’s decision-making power. Table 1 presents the main themes and sub-themes that were identified in the analysis.

### Facilitators of men’s involvement

Facilitators of men’s involvement refer to the various factors that reinforce men’s involvement in maternal and child health.

#### Recognition of impact of men’s involvement in maternal and child health

One of the main facilitators of men’s involvement in maternal and child health was men’s recognition of the benefits of their involvement. Men themselves expressed that their participation was important because they were provided with information that would help them protect the baby from contracting HIV, encourage women to eat food that would promote the health of the unborn child and recognise signs that could lead to maternal mortality. One man in an all-male focus group discussion said:

*P: When it is the first time, we learn together with them. They tell us that expectant women are not supposed to do very tiresome work. They need to eat different food groups and they mention those food groups like meat, eggs, beans, vegetable and fruits like bananas, mangoes and papayas.*

It was clear from the findings that when men received information on nutrition during their partner’s pregnancy, they tried to ensure that their partners accessed the necessary food for the growth and development of the baby. Men mentioned that the hospitals encourage them to provide milk to women milk when they are pregnant.

*P: Then we try as much as possible to give her milk at home. We get milk on loan from fellow villagers and the debt is paid on monthly basis. A bottle of coca cola is worth MWK300, so we buy every day and at the end of the month we pay MWK 3000. It is very helpful.*

*{Man in a focus group discussion}.*

#### Pride

Pride was another factor that facilitated men’s participation. The men took pride in identifying their own community as the ‘number one village in terms of safe motherhood’. One man said:

*P: When people from the government are coming to research on safe motherhood, they come to this community.*

Women also mentioned that when men attended antenatal care, they had the opportunity to see other healthy children. This motivated them to try to make sure that they too provided their wives with the necessary resources to ensure a healthy pregnancy outcome.

#### Advocacy

Another facilitator of men’s involvement in maternal and child health was advocacy. Both informants and community members mentioned that NGO advocacy and messaging played an important role in encouraging men to participate in maternal and child health. Women mentioned that radio messages helped to motivate men to participate in maternal and child health. One man in a focus group discussion said:

*P: The gender issue is triggering these acts. Before, when we went to the farm with our parents after farming our mother would carry a lot of things while our father would carry nothing. But these days, because of advocacy, we have realised that we were not treating women [*sic*] nicely. So we help with the household chores, but before, that was not happening.*

Gender messaging also made men realise that men needed to take a more active role in the household and assist their wives with cooking, cleaning and child care. Concern, Care Universal Malawi, Catholic Relief Services and The Hunger Project were mentioned by men and women participants as playing a particularly important role in advocating and providing messaging for men’s involvement.

#### Incentives and disincentives

A further facilitator of men’s participation in maternal and child health that was identified was incentives and disincentives that either encourage men’s involvement in maternal and child health or dissuade them from acts that were detrimental to maternal child health respectively. Incentives and disincentives were reinforced by both hospitals and Traditional Authorities. Clinics in Malawi have developed an incentive and disincentive system to motivate men to attend antenatal care along with their partners. Women attending antenatal care with their partners are attended to first. For women who have travelled a great distance to get to the clinic and have left fields unattended to make their clinic appointment, being attended to in a timely manner is important. Many women lose an entire day’s work in order to access healthcare, which for a small holder subsistence farmer is considered a great loss. The order in which women are served at the antenatal clinic should thus be understood within this context. One woman said:

*P: Let’s say you reach your fourth month and you tell your husband that you should go to antenatal clinic. You are at an advantage because even if you get there late, you are given place at the front of the queue. You end up finishing quickly. I’m talking from experience because when I went to the clinic with my husband we were fourth in the queue and they moved us up.*

Another woman said:

*P: The first thing is that a woman who has gone with the husband is attended to first. The other women seeing that plead with their husbands to go with them the next time so that they too are attended to quickly.*

If women attend antenatal care without their partners, they are either served last or not provided with services until they return with their partner. In support of the clinics, Traditional Authorities have also developed a set of bylaws to reinforce the incentives and disincentives. If a woman was abandoned by her partner, her family serves as witness to the chief who will then provide her with a letter excusing her from attending antenatal care with a partner. This letter is taken by the women to the clinic.

#### Male champions

Male champions are responsibile for delivering messages of safe motherhood to other men within a community. Secret men or lead fathers in care groups also facilitate men’s involvement. Secret men and secret women travel from house to house discretely encouraging men to be involved in maternal and child health and to attend antenatal care. In Malawi, issues of sexual and reproductive health have traditionally been considered taboo to discuss in public. Also, the sensitivity of some of the issues discussed requires that conversations be conducted discretely. Secrecy is observed because, in many cases, secret men and in particular secret women, discuss issues sensitive topics around sexual and reproductive health, which may include family planning, HIV and unwanted pregnancy. Both informants and community members suggested that male champions played an important role in facilitating men’s participation in maternal and child health. One woman said:

*P: The nurses are the ones that write a letter to the chief to say that the woman is not being escorted to antenatal clinics by her husband. That means the chief will go and speak to the husband. But there are also secret men who can go and visit them.*

*{Woman in a focus group discussion}.*

Lead fathers in care groups also assist in encouraging men to get involved in maternal and child health. Care groups are committees that are setup to provide maternal and child health, nutrition and care information to households. They consist of 6–15 participants. Men have been integrated into this system to encourage other men to attend antenatal care and to support their wives during pregnancy. In both the case of the secret men and the care groups, it was felt by the men that they would be in a better position to motivate other men to be involved in maternal and child health, taking into account gendered social dynamics that cause men to be more apt to listen to other men than to women.

### Barriers to men’s involvement

Although men’s involvement in maternal and child health has seen some success in the Traditional Authority where the study was conducted, not all men are willing to participate in maternal and child health. Barriers to men’s involvement refer to the various factors that prevent men from being involved in maternal and child health. Some women participants expressed that their partners were not involved in their pregnancy. Some informants offered different perspectives as to why men were constrained to participate in maternal and child health. These constraints included; socio-cultural beliefs, stigmatisation of men involved in maternal and child health and opportunity costs associated with attending antenatal care.

#### Socio-cultural beliefs

Regardless of the involvement of the Traditional Authorities, socio-cultural beliefs that prevent men from participating in maternal and child health were prevalent. Female participants expressed that not all men were able to understand the importance of men’s participation in maternal and child health and continued to see pregnancy as a woman’s responsibility.

The male participants expressed that men who are not able to appreciate the importance of participating in maternal and child health are likely still being influenced by traditional beliefs. Informants expressed that women too are influenced by socio-cultural beliefs that prevent them from allowing the men to perform typically ‘female-related’ tasks. For example, one informant said:

*P: Because in the village, they don’t allow a man to cook. Even if the woman is sick, they will invite someone to come and cook. Even that woman will not be very free to have her man come and cook. So when you talk of behavioural change, it has to be both men and women.*

#### Stigmatisation of men involved in maternal and child health

Stigmatisation is another barrier closely related to socio-cultural beliefs. According to the female participants, stigmatisation of men who participated in what they considered to be typically ‘female activities’ endures. The women expressed that even in situations where their partners wanted to be involved in maternal and child health, men were ridiculed or mocked for engaging in ‘women’s work’.

*P: There are those who understand, but because of chatting with their friends when they see them doing these things, they tell them, you are weak. They have given you medicine.* 1 *You’re helping carry the garden tools and sweeping? That is a woman’s job. Instead of continuing to do what he has learned, he becomes embarrassed and says, I shouldn’t do this. My friends will laugh at me.*

*{Woman in a focus group discussion}.*

#### Opportunity costs associated with attending antenatal care

Another barrier that was expressed mainly by the informants was that there were high opportunity costs associated with men’s involvement in antenatal clinics. The time spent travelling to and from the health centre could have been used for farm work, or other income generating activities. The lack of a conducive environment for men’s participation further exacerbated men’s frustration with the time lost. Information presented at the health facilities, for example, was often targeted at women and the songs sung as part of the education sessions might be offensive to men. An excerpt from one of the songs sung at one of the health care centers visited is translated as:

*He has drunk tameki,* 2

*He has drunk tameki,*

*Because of having too many children.*

*He has gone to South Africa,*

*He has gone to South Africa,*

*Because of having too many children.*

Some informants mentioned that at some health facilities, there are no activities to keep men occupied while women receive attention. Some informants were also concerned that when men were present, women were not able to speak freely during HIV testing and counselling sessions and that men’s involvement might prevent women from disclosing information vital to the growth and development of the baby.

### Limitations to men’s involvement

Besides these barriers, there are also some limitations to the way in which interventions for men’s involvement in maternal and child health were designed in Malawi. Limitations to men’s involvement in maternal and child health referred to factors that may have unintended consequences on the desired outcome of programmes.

#### Discriminating against women

There were some mixed responses to the incentives and disincentives used to encourage men to participate in maternal and child health. Although some of the informants and community members alike felt that withholding services from women who attended antenatal care without a partner was important because it forced men to attend antenatal care, others felt that women were being punished for offences that were not within their control.

*P: Now they have shifted blame on women. Now they are saying that whoever does not bring their husbands must be punished. That is what is happening. That policy or bylaw punishes women. It puts the responsibility of involving men on the women. It punishes women.*

*{Female informant in an individual interview}.*

Even in cases where treatment was not withheld, there was some concern that bylaws gave preferential treatment to women with husbands and discriminated against women who did not have partners with whom to attend antenatal care. One woman said:

*P: So when they go there they ask “those who have come with their husbands come first”. Then those who are pregnant but didn’t have husbands, even though they came early, stay behind.*

Another woman said:

*P: So if you come with a spouse at whatever time, you will be the first to be attended to. Women felt that it was not good for them because they would wake up early in the morning, they would come to the clinic and stand in the queue, and then someone comes with a spouse and they are attended to, it was very unfair.*

Unmarried women were further marginalised by these bylaws because they are expected to pay a fine to the chief. Women who had been abandoned by their partners or fell pregnant out of wedlock were fined. It was reported during the discussions that some women have found ways around paying these fines. Informants reported that some women replicated letters from the chief that exempted women from attending antenatal clinics with their partners, although the chiefs insisted that they write the letter in such a way that it is not replicable. Others noted that some women pay men who they find loitering around the clinic to pose as their partners, negating the intended benefit of men’s attendance of antenatal clinics.

#### Reinforcing men’s decision-making power

Men’s involvement in the area where the study was conducted does not appear to always have an impact on gender equality. The findings suggest that in areas where men’s involvement programmes are not complemented by gender equality activities, such as joint family planning counselling, men’s involvement is used as a tool to bypass gender norms. For example, although men attend antenatal care because of the pressure that they received from the Traditional Authorities and the clinics, there was not necessarily equal decision-making in the home. Some men still refrain from helping with housework – which they consider to be ‘women’s work’. Although men were encouraged to attend antenatal care, some men continued to regard child care as ‘women’s work’.

In some areas where gender sensitisation campaigns have been complemented with advocacy on men’s involvement, men have been able to apply lessons to other aspects of life.

*P: These days, because of advocacy, we have realised that we are not treating women [*sic*] nicely. They are also the same as us…so we help them with the household chores. But initially, this was not happening.*

*{Man in a focus group discussion}.*

Another woman in a focus group discussion said:

*P: It is now when they brought in gender to say that when coming from the field, the man has to carry his hoe. When they get home, the man has to be doing other work like sweeping the surrounding while the woman is cooking. This time, things are just ok. You just make sure that you cook relish when you are going out and he will cook nsima.*

However, in most cases, men’s involvement does not go beyond men’s attendance of antenatal clinics. The bylaws were limited to only incentivising attendance of antenatal clinics. Socio-cultural beliefs related to inequality and stereotyping with regard to gender roles and responsibilities remained prevalent, regardless of men’s participation in antenatal clinic visits.

Although attendance of antenatal clinics was deemed important for maternal and child health, once the child is born the responsibility of child care and housework often reverts to women. For example, one woman in a focus group explained that men were forced to go to antenatal clinics because they have to be tested for HIV, but they do not attend postnatal clinics.

## Discussion

While men’s involvement in maternal and child health in Malawi has been often understood to mean men’s involvement in antenatal clinics to reinforce PMTCT, this study found that men’s involvement in maternal and child health in rural Central Malawi also includes men’s active roles in nutrition, child care and other household responsibilities. Often men’s involvement occurs when women are pregnant or ill, but in some instances, men’s involvement remained consistent, adopting more equitable roles. Men expressed that their involvement protected their unborn child by encouraging mothers to attend antenatal clinics and meeting their dietary requirements. Their involvement also included providing emotional support to women and recognising signs that could lead to maternal and child mortality. Men expressed that they were also responsible for providing financial support to acquire food and other necessities required during the delivery.

Advocacy from NGOs, media, male champions as well as traditional leaders plays a critical role; not only facilitating men’s involvement but also in overcoming gender stereotypes and reinforcing gender equality. The continuous messaging on men’s involvement in gender equality in conjunction with the bylaws, created an environment where stigmatisation was reduced and men were free to actively be involved in maternal and child health. However, in cases where the bylaws and d/incentives were applied without supporting activities from the NGOs and male champions, men’s involvement was limited to attending antenatal care. This suggests that men’s participation in antenatal care alone is not sufficient for addressing institutionalised gender inequalities. The study findings are consistent with August et al. [34] who suggested that when men’s involvement programmes are implemented without complementary activities such as decision-making and gender equality sensitisation programmes, men’s involvement simply reinforces gender inequalities.

The role of the traditional leaders in enforcing men’s involvement is a notable finding for facilitating policy implementation, particularly regarding behaviour change. Although much controversy surrounds traditional leaders in Malawi because of nepotism in resource allocation, their role in the context of men’s involvement in maternal and child health has been important in influencing adherence to attendance of antenatal clinics. Their commitment to improving maternal and child health is evident through the bylaws established. While the bylaws enable traditional leaders to take advantage of unmarried women who are subjected to paying fines, the involvement of traditional leaders has advanced the agenda of proponents of men’s involvement in maternal and child health. This finding emphasised the need for healthcare and nutrition practitioners and policy-makers to involve traditional leaders strategically in the development of policy and implementation.

It is important to note that the bylaws created by traditional leaders, although not legally binding, infringe upon women’s constitutional and human rights. The Constitution of Malawi [35] states that all people have the right to health services. Therefore, when health care facilities withhold services because women have come without their partners, they are in breach of the Constitution. Furthermore, women travel long distances to attend antenatal care and serving them last could serve as a disincentive for their continued attendance of antenatal clinics.

The findings of our research are consistent with literature that suggests that socially constructed norms around gender continue to hinder men’s involvement in maternal and child health. For example, both Kululanga et al. [9] and Aarnio et al. [10] suggest that pregnancy is typically a woman’s domain and that men do not feel that they should be involved. However, our findings differ slightly in that undertones of change are evident. For example, some men are gradually becoming active in nutrition activities relating to maternal and child health, another domain which was previously associated with women. In situations where men’s involvement programmes have been implemented with complementary gender equality activities in Malawi, men were often responsible for providing food and in some cases even preparing food.

Although there is a need for joint counselling sessions for couples, there is also clearly a need for separate activities for men and women during antenatal clinic visits. Women have historically owned the antenatal care domain and the environment is one in which they have space to share and express themselves. The design of men’s involvement programmes should consider this need and provide a safe space for couples to engage but also provided separate spaces for each of the sexes to express themselves. For example, men could have separate spaces where they could engage with one another on their role in the household.

Men’s involvement in maternal and child health has implications for maternal and child nutrition. When men attend antenatal clinics, they are provided with information that encourages them to appreciate the importance of their participation in maternal and child nutrition. They are also able to recognise the importance of allocating resources to diverse foods and as this study showed, even go out of their way to obtain loans to ensure their partners meet their dietary requirements. Men’s attendance of antenatal clinics provides an opportunity for exposure to messages that enable men to make more informed decisions on food choices, food purchases, food production and allocation of resources toward food. Men’s involvement in child care activities, as well as household activities, suggests that messaging on food preparation and infant and young child feeding need to be gender sensitive with particular attention to how men receive messages. Similarly, images in the media and other public messages need to be gender sensitive and consider men’s involvement in the presentation. Policy-makers often make the assumption that only women are and can be involved in nutrition. As a result, they too may perpetuate socially proscribed gender roles. Based on our study findings, we suggest that not only are there benefits to involving men, but men are already getting involved in nutrition. Therefore, policy-makers need to be aware of men’s involvement in maternal and child nutrition and promote an environment that is conducive for and facilitates men’s involvement.

## Conclusion

This study is one of the few to have focused on analysing men’s involvement in maternal and child health interventions, with specific attention to the implications of men’s involvement in maternal and child health for nutrition policy. The study findings identify several facilitators of men’s involvement in maternal and child health. Although socio-cultural barriers remain, evidence of change is clear. However, limitations need to be addressed to ensure that men’s involvement activities do not infringe upon women’s human rights.

Policy-makers need to ensure that they create a conducive environment for men’s involvement in maternal and child health by providing support services that motivate men to want to be involved. Interventions that support men’s involvement in maternal and child health should not undermine women in decision-making but rather enhance equitable decision making in the household. Policy-makers should recognise that gender dynamics at community level are changing and that policies should be respond to these changing gender dynamics.

The study concludes that stakeholders involved in maternal and child health did not respond to emerging gender concerns that arose from the men’s involvement in maternal and child health approaches. For example, bylaws intended to motivate men to attend antenatal care by withholding services from women who did not attend antenatal care with their partners discriminated against women. Service providers cannot withhold critical treatment. Policy-makers in Malawi should ensure that men and women have the knowledge and the capacity to demand their rights in non-confrontational ways as partners and stakeholders in their own treatment.

While men’s involvement interventions are important for maternal and child nutrition, the design of these interventions undermine gender equality. Consequently, the real constraints that confront men and women in terms of accessing nutritious food are not addressed. More supportive programmes are needed in all districts in Malawi to promote men as agents of change. Nutrition policy interventions need to complement activities in health by providing information on joint decision-making in resource allocation for food and food production choices.

## Footnotes

1 In rural communities in Malawi, it is common for people to be superstitious about certain behaviours that appear uncommon. As such, people often make the assumption that when a man is engaging in work that is typically considered women’s responsibility, he has either been bewitched or has been given some potion to change his behaviour.

2 *Tameki* is the brand name of a rat poison in Malawi
